# Ultrasound molecular imaging: insights into cardiovascular pathology

**DOI:** 10.1007/s12574-020-00463-z

**Published:** 2020-02-13

**Authors:** Koya Ozawa, Jonathan R. Lindner

**Affiliations:** 1 Knight Cardiovascular Institute, Oregon Health & Science University, Portland, OR 97239 USA; 2 Cardiovascular Division, Knight Cardiovascular Institute, The Oregon National Primate Research Center, Oregon Health & Science University, 3181 SW Sam Jackson Park Rd, Portland, OR 97239 USA

**Keywords:** Molecular imaging, Contrast-enhanced ultrasound, Targeted microbubbles, Cardiovascular disease

## Abstract

Similar to what has already occurred in cancer medicine, the management of cardiovascular conditions will likely be improved by non-invasive molecular imaging technologies that can provide earlier or more accurate diagnosis. These techniques are already having a positive impact in pre-clinical research by providing insight into pathophysiology or efficacy of new therapies. Contrast enhanced ultrasound (CEU) molecular imaging is a technique that relies on the ultrasound detection of targeted microbubble contrast agents to examine molecular or cellular events that occur at the blood pool-endothelial interface. CEU molecular imaging techniques have been developed that are able to provide unique information on atherosclerosis, ischemia reperfusion injury, angiogenesis, vascular inflammation, and thrombus formation. Accordingly, CEU has the potential to be used in a wide variety of circumstances to detect disease early or at the bedside, and to guide appropriate therapy based on vascular phenotype. This review will describe the physical basis for CEU molecular imaging, and the specific disease processes for the pre-clinical translational research experience.

## Introduction

**Fig. 1:**
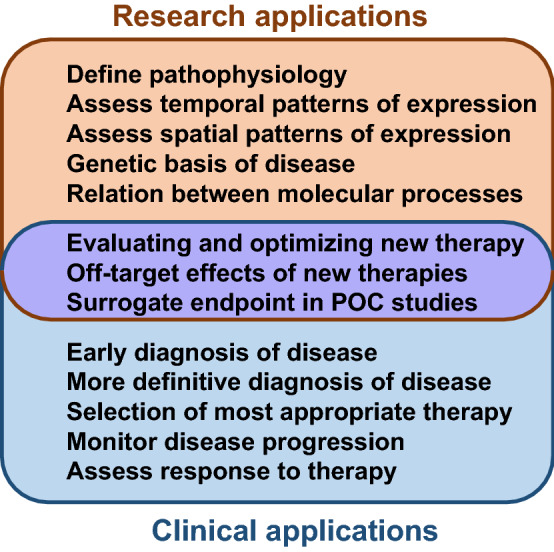
Applications for noninvasive molecular imaging which span from research (top), clinical applications (bottom) and dual research/clinical applications (middle)

Non-invasive in vivo molecular imaging techniques have been developed to assess molecular or cellular phenotype in animal models of disease and in humans. These methods provide both temporal and spatial assessment of disease-related molecules, and are particularly impactful when they are combined with conventional methods for imaging function, structure and flow. There are many reasons from both the research and clinical perspective for the development of molecular imaging technology (Fig. 1 )[1–3].In preclinical research, molecular imaging is now considered to be a valuable asset for investigating spatial and temporal patterns of molecular pathophysiology, and for rapid evaluation of on-target and off-target effects of new therapies. In the clinical realm, molecular imaging could be used for earlier or more rapid diagnosis of disease, for better risk stratification, and for achieving the goals of precision medicine by selecting the most appropriate drugs based on molecular phenotype and then assessing response to those therapies.

There are many different approaches to molecular imaging [4]. One of the most common approaches has been to create novel targeted imaging probes which can be detected by noninvasive imaging techniques such as radionuclide imaging (e.g. positron emission tomography [PET]), magnetic resonance imaging (MRI), ultrasound or optical imaging [4–6]. For the diverse array of imaging technologies, some of the technical characteristics that differentiate the various pattern of molecular imaging are provided in Table 1. There are major differences between probes according to kinetics, sensitivity, specificity, distribution, toxicity, and cost for the different imaging technologies.

**Table 1:** Characteristics of methods used for molecular imaging

	Ultrasound	SPECT	PET	MRI
Spatial resolution	+ + +	+	+ +	+ + + +
Temporal resolution	+ + + +	+ +	+ + +	+
Sensitivity	+ + +	+ + + +	+ + + +	+
Target distribution	Intra-vascular	Diffusible	Diffusible	Leakable
Affected by shear	Yes	No	No	Possibly

Contrast-enhanced ultrasound (CEU) molecular imaging relies on the selective targeting and detection of encapsulated microbubbles (MBs) or other acoustically active micro or nanoparticles which are retained in tissue on the basis of their ability to bind to molecules. CEU has several unique advantages compared to other forms of molecular imaging. CEU images are able to be obtained very rapidly, within minutes of intravenous injection; and the lack of any need for extensive post-processing provides almost immediate information to the clinician. When compared to other technologies, CEU is balanced with regards to sensitivity and spatial resolution, which are often divergent when comparing modalities. CEU is also portable, inexpensive, and does not involve radiation; all of which are practical considerations for the use of the technology for population screening or even point-of-care assessment of patients. However, CEU is limited in terms of its molecular targets since conventional platforms for CEU using microbubbles can provide information only on events within the vascular compartment. In this review, we will focus on some of the basic principles for CEU molecular imaging and highlight most pertinent clinical applications where CEU molecular imaging may have a major impact.

## Targeted ultrasound contrast agents

Conventional ultrasound contrast or enhancing agents are composed of MBs which contain a high molecular weight inert gas such as perfluorocarbons or sulfur hexafluoride, and have a shell composed of lipids, protein (albumin), or biocompatible polymers. The size of MBs that are typically used for cardiovascular applications in humans is generally in the range of 0.5−5 μm in diameter, which permits their intravenous injection and unimpeded passage through the microcirculation of the pulmonary, coronary and peripheral microcirculations [7, 8]. The signal generation from these agents occurs through either stable cavitation (volumetric oscillation without destruction) or inertial cavitation (destruction and dispersion/dissolution of gas core) within the pressure peaks and nadirs of the ultrasound field [9, 10]. Both stable cavitation and inertial cavitation leads to emission of ultrasound signal and ultrasound energy peaks at harmonic frequencies, with inertial cavitation producing strong broad-band signals but also immediate or rapid destruction of agent [10–12].

**Fig. 2:**
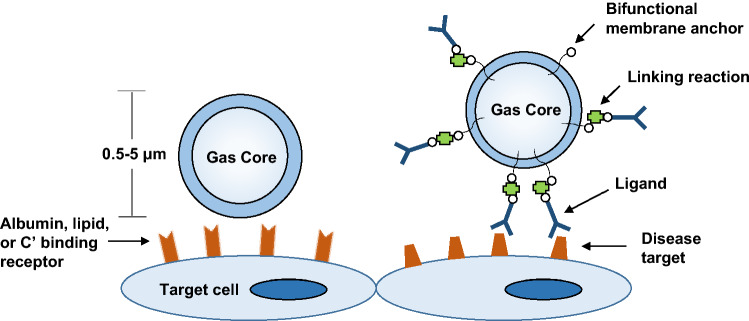
Approaches used for targeting of microbubble or other ultrasound enhancing contrast agents to a target cell.* C’* complement proteins

CEU molecular imaging is commonly performed using targeted MBs that have been retained by adhesion to specific molecules or cells that are within the vascular compartment. Targeting of MBs agents has been accomplished by two general approaches (Fig. 2 ). A simple approach for targeting relies on non-specific retention of MBs by their binding to endothelial cells or specific leukocyte populations that have undergone activation with subsequent increase in surface receptors that recognize MB shell component. Several mechanisms are involved in these types of interactions, the most common of which involves opsonization, whereby serum complement proteins that deposit on the MB surface are recognized by complement receptors on leukocytes or on endothelial cells [13–15]. The composition of the MB shell composition, including net charge and the presence of polymeric surfactants such as polyethylene glycol are important determinants MB interaction with cells. The presence of phosphatidylserine, a phospholipid that when on the outer leaflet of cell membranes acts as a signal of senescence or apoptosis, in the MB shell can enhance their attachment to activated cells through opsonization [15]. Non-specific interactions with activated leukocytes and endothelial cells can be leveraged for generally detecting the presence of inflammatory states rather than any one particular molecular pathway.

**Fig. 3:**
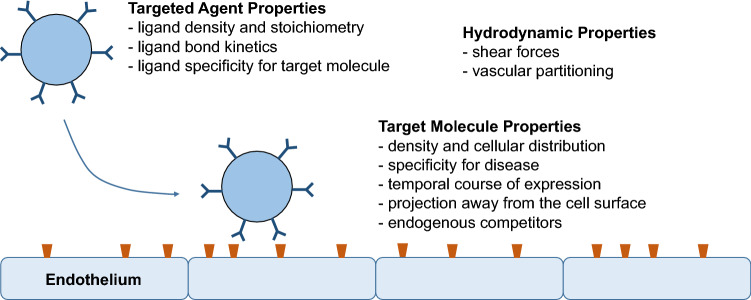
Determinants of targeted microbubble retention in areas of disease. Factors that influence microbubble retention are separated into contrast agent variables, target molecular variables, and hydrodynamic properties within the vascular compartment

A more specific approach for targeting of MBs is to conjugate disease-targeting ligands (antibodies, peptides, glycoproteins, small molecules, etc.) to the surface of MBs. Targeting ligands can be ligated to the MB surface using any number of chemical conjugation strategies. The density of targeting ligands can reach several thousand per square micrometer of surface area. The performance of any MB bearing a targeting ligand is determined by many factors (Fig. 3). Some of the most important determinants include specificity of the targeting ligand to the molecule of interest, surface density of the targeting ligand and the molecular target, bond kinetics under different shear conditions, off-target non-specific binding, and presence of endogenous inhibitors (decoys). It is also important to select the most appropriate target molecule based on its relevance or specificity to the disease of interest, and its temporal pattern of expression.

## Methods for targeted CEU molecular imaging

There are many methods that have been developed to specifically detect the non-linear signal that arises from stable cavitation of MB contrast agents in the blood pool [9]. Studies have confirmed that signal generated from microbubbles and their acoustic lability are not substantially influenced by their ligation to cells or even their internalization by phagocytic cells [16, 17]. Hence, the ultrasound system settings for performing molecular imaging are not substantially different than for conventional contrast echocardiography. However, there are specific protocols for extracting molecular imaging information. Generally, targeted MBs are administered as an intravenous bolus injection and their retention in tissues are registered in one of two ways. One approach involves imaging after a wait time of 5–10 min to allow clearance of MBs from the circulating blood pool, the rate of which depends on the tissue of interest, the animal species and the type and dose of MBs. Imaging after signal intensity for tracer retained is measured after the majority of freely circulating MBs have been cleared. The signal on the initial frame upon resumption of imaging reflects the total tissue signal enhancement of both retained MBs and the trace amounts of freely circulating MBs, the latter of which can be determined and digitally subtracted by destroying all MBs with high-power ultrasound then observing for the amount of signal returns from freely circulating agent [18]. An alternative methods that can be used when differences in blood flow markedly alter the number of MBs transiting through a tissue is to use transfer kinetics to estimate the retention fraction. For this approach, signal intensity is continuously measured after a venous injection which allows deconvolution of two curves: one representing free tracer which rises rapidly and gradually decays, and the other representing the integral of the first curve which represents MBs that enter but are permanently retained [19].

## Atherosclerosis imaging

The pathophysiology of atherosclerosis is complex and changes over the decades of disease development. However, a process common in both early disease development and late complications involves vascular inflammation [20]. The ability to image inflammation may provide clinical utility for detecting high risk at an early stage or for stratifying patients with known advanced disease to specific anti-inflammatory therapies. In the research arena, molecular imaging of pro-inflammatory pathways may be useful for identifying new therapeutic targets or for evaluating the efficacy of drugs in development for either palliating high-risk atherosclerotic lesions or for arresting disease at an early time point. Ultrasound molecular imaging is particularly useful for the non-invasive assessment of endothelial pro-inflammatory changes, such as the expression of leukocyte adhesion molecules, since MBs detect adhesion molecules that are actively expressed within the vascular lumen. Although there are many potential targets for molecular imaging in atherosclerosis that could be useful for clinical or research purposes, the target must be governed by the intended clinical use and the stage of disease. For example, the assessment of future risk of progressive disease in an individual without known disease is likely to rely on the detection of early inciting events such oxidative stress, lipid accumulation, or endothelial cell adhesion molecule expression that participate in leukocyte recruitment. Detection of the “vulnerable plaque” or “vulnerable patient” in advanced disease could include many different targets within the plaque, most of which are not accessible to traditional MB constructs.

**Fig. 4:**
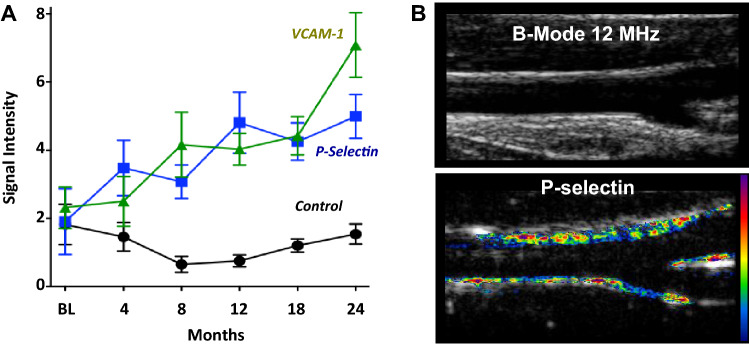
**a** CEU molecular imaging data from the carotid arteries of rhesus macaques at baseline (BL) and after starting a Western-style High-Fat diet (HFD). Molecular imaging signal is shown for microbubbles targeted to endothelial VCAM-1, P-selectin, or control microbubbles. Signal for P-selectin and VCAM-1-targeted MBs were significantly higher (*p*<0.05) than for control MBs beginning at 8 months. **b** Examples of non-contrast 2-D B-mode imaging (top) and CEU molecular imaging of P-selectin (bottom) demonstrating enhancement of the vascular endothelium (color-coded with scale to right) after 12 months of HFD. Modified from Chadderdon S et al., Circulation 2014;129:471

For the detection of aggressive atherogenesis, CEU molecular imaging of adhesion molecules such as selectins, intercellular-adhesion molecule-1 (ICAM-1), and vascular cell adhesion molecule-1 (VCAM-1) has been applied in murine models of disease [21–23]. These molecules are involved in the recruitment of innate immune cells into plaque. Hence they have been found to provide one of the earliest markers for detecting the onset of aggressive atherogenesis [21]. Carotid CEU molecular imaging of P-selectin and VCAM-1 performed in non-human primates has been shown to detect the onset of early stage of endothelial activation in response to high-fat diet and obesity, prior to any change in carotid intima-media thickness (Fig. 4 ) [24]. CEU is also able to detect prothrombic vascular events associated with acute vascular events, such as fibrin, exposed tissue factor, and platelet aggregation [22, 25–27]. However, the clinical utility of strategies for imaging individual “vulnerable plaques” remains undecided. More recently, molecular imaging of excess endothelial Von Willebrand factor and secondary vascular platelet adhesion has provided new evidence for the role of the platelet adhesion in early atherogenesis, in accelerated plaque growth after an ischemic event, and in chemotherapy-related arterial events [28–30]. This approach to atherosclerosis has not only provided a better understanding of the pathophysiology of aggressive atherosclerotic disease, but has been used to evaluate the efficacy of new therapies as well [29–31].

## Ischemic memory

In patients presenting with Acute Coronary Syndrome (ACS), the clinical diagnosis generally relies on history information, serologic markers such as troponins, and electrocardiogram (ECG). It is widely recognized that many patients do not have classic angina symptoms, and many of those with ACS do not have diagnostic changes on the ECG [32]. In those with unstable angina, there are limitations in both sensitivity of ECG and serologic markers. Diagnostic tools for rapidly evaluating both the presence and the spatial extent of recently resolved myocardial ischemia improve the care of patients seen with suspected unstable coronary syndromes.

**Fig. 5:**
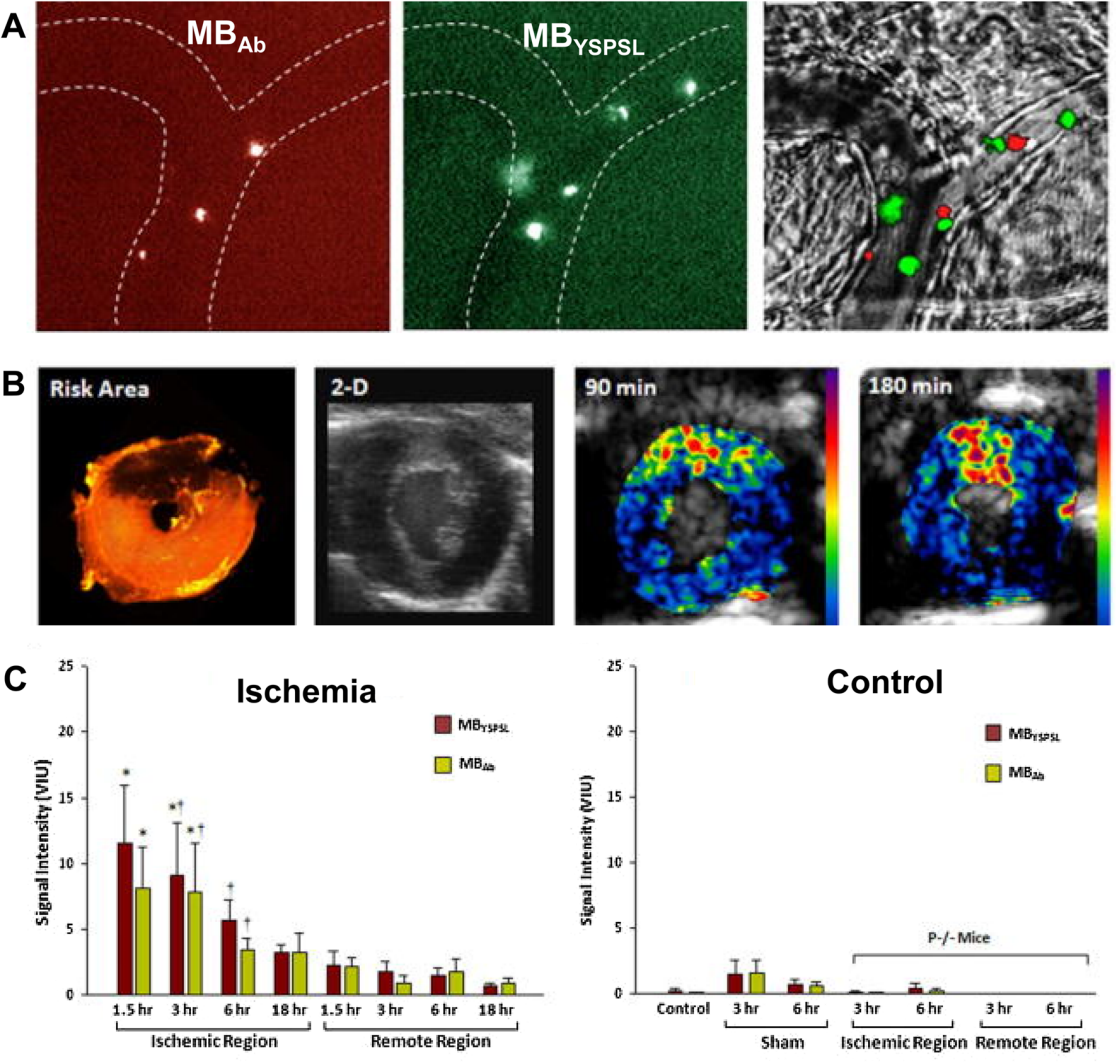
Molecular imaging of recent ischemia with P selectin targeted microbubbles. **a** Intravital microscopy from a muscle undergoing ischemia reperfusion injury showing venule (outlined in dashed lines) retention of differentially-labeled red- or green-fluorescent labeled microbubbles targeted to selectin by a selectin antibody (MB_Ab_) or a small biomimetic glycoprotein (MB_YSPSL_). Dual fluorescent overlay with transillumination are shown at right. **b** Short-axis images from a mouse heart showing the post-mortem microsphere-defined LAD risk area, 2-D non-contrast transthoracic echocardiography, and color-coded images obtained during molecular imaging of P-selectin after intravenous injection of (MB_YSPSL_) at 90 min or 180 min after LAD ischemia–reperfusion. **c** Signal enhancement from the two different P-selectin-targeted microbubbles from the post ischemic anterior wall several hours after brief temporary ischemia, and the remote posterior myocardium (left graph). Data are also shown from control experiments (right graph) involving normal animals (controls), sham-treated animals, and post-ischemic P-selectin-deficient (P-/-) mice. Adapted from Davidson BP, et al., J Am Coll Cardiol 2012;60:1690

CEU molecular imaging has been developed to assessing ongoing or recent, but resolved, myocardial ischemia at the patient bedside. For such “ischemic memory” molecular imaging, MBs have been targeted to activated endothelium by either ligands for the endothelial selectin family of adhesion molecules which are expressed rapidly after ischemia, or by including phosphatidylserine (PS) into the shell of MBs (Fig. 5 ). Targeting of selectins on the endothelial surface has been validated in murine and non-human primate models of myocardial infarction, and allows imaging of recently ischemic myocardium for many hours after its resolution [33, 34]. The simpler approach of using PS as a targeting moiety is based on its ability to amplify opsonization, thereby representing an easier path to clinical translation. This approach has been used in canine models and murine models of resolved myocardial ischemia, and appears to as effective as selectin targeting [35, 36].

## Thrombus imaging

Thrombosis has a key role in many pathophysiologic processes in cardiovascular disease. There are many different anti-thrombotic and antiplatelet therapies designed to inhibit thrombus formation or to accelerate dissolution of clot [37]. Because any antithrombotic therapy is associated with risk of bleeding, clinicians must have a high level of confidence that a patient will benefit from their use. Accordingly, methods for non-invasively imaging thrombus in the cardiac chamber, in large vessels or in the microcirculation will likely have a role in selecting the most appropriate therapy in patients based on refining risk-to-benefit ratio.

As mentioned above, prothrombotic processes involved in atherogenesis or complications of atherosclerosis have been imaged with CEU molecular imaging of VWF, platelets, fibrin and tissue factor. Targeted imaging using similar agents has also been applied to detect large thrombi within vessels or the cardiac chamber, or microvascular thrombosis such as that occurs with post-MI no-reflow phenomenon. The relative role each for each of these individual agents is influence by whether the thrombotic process of interest is fibrin-rich or platelet-rich; and the shear forces in the chamber/vessel involved. Fibrin-targeted agents have been shown to produce enhancement not only in areas of vascular mechanical injury, but also on ventricular thrombi in large animal models [22, 38]. Agents targeted to platelet-mediated processes have, in particular, been shown to provide unique information to justify use in certain circumstances. One example is that CEU with MBs targeted to GPIIb/IIIa which mediates platelet aggregation has been used to monitor the efficacy of thrombolytic therapy in real-time [27]. Recently, CEU molecular imaging with targeted MBs for platelet GPIbα and VWF have been applied to rapidly evaluate microvascular no-reflow phenomenon in rodent model of post-MI reperfusion, and the efficacy of new approaches to improving reflow.[39]. Additionally, CEU molecular imaging for platelet GPIbα and VWF have been developed to reveal a new form of vascular toxicity for the third-generation tyrosine kinase inhibitor ponatinib that involves VWF-mediated platelet adhesion and a secondary microvascular angiopathy that produced ischemic wall motion abnormalities [30]. Finally, it is also important to remember that the targeting of microbubbles to thrombus may aid in ultrasound mediated therapy for thrombotic disorders such as delivery of fibrinolytics or with sonothrombolysis [40–42].

## Summary and future for CEU molecular imaging

Whether using molecular imaging for clinical diagnosis or for research purposes, the selection of the most appropriate imaging technique requires a consideration of the advantages and disadvantages for each approach. Because CEU unlike other forms of molecular imaging uses pure intravascular contrast agents, the most common cardiovascular disease-related processes imaged include endothelial activation, leukocyte recruitment, angiogenesis, and thrombus formation. In general, MRI and CT are considered to have the highest spatial resolution, while radionuclide imaging (PET, SPECT) tends to have the highest sensitivity and dynamic range for detecting tracer. CEU molecular imaging provides a good balance between spatial resolution and sensitivity. CEU also has the advantage of being able to provide near simultaneous structural and molecular imaging. With regards to temporal resolution and speed of acquisition, CEU can be performed with rapid acquisition times of less than 10 min, while commonly used PET/SPECT and MR protocols require anywhere from just under an hour to a day between contrast injection and imaging. Ultrasound is the only technique that can be used for bedside diagnosis and is the lowest cost for all advanced imaging modalities.

With all of the considerations above in mind, in the clinical setting, CEU molecular imaging is an ideal approach when rapid point-of-care decisions need to be made, such as ischemic memory, or for use as a screening technique in large populations, such as in atherosclerotic risk assessment. However, the translation of these technologies to humans continues to be challenging. One major obstacle to clinical adoption is the complex nature of regulatory agencies that oversee the Good Manufacturing Processes (GMP) including Chemistry, Manufacturing and Control (CMC) validations. Another major hurdle has been the difficulties in funding large trials that will be needed to show not only efficacy, but changes in outcome based on incremental value in large clinical trials. Recently, a microbubble agent targeted to Vascular Endothelial Growth Factor (VEGF) receptors has been translated to humans for potential use in cancer detection. However, these trials were small exploratory trials without controls [43, 44], and efficacy in terms of signal enhancement and sensitivity was low [44]. For cardiovascular applications, a commercially-produced MB agent containing phosphatidylserine has shown promise for ischemic memory imaging in pre-clinical models [36], and is currently being assessed in a clinical trial (NCT03009266). Although the translation of CEU molecular imaging from pre-clinical to clinical trials is just beginning, the technology is already established in the research setting and is used to understand pathophysiology of cardiovascular disease and efficacy of new therapies.
